# Multiomic Characterization of High-Grade Serous Ovarian Carcinoma Enables High-Resolution Patient Stratification

**DOI:** 10.1158/1078-0432.CCR-22-0368

**Published:** 2022-06-13

**Authors:** Robert L. Hollis, Alison M. Meynert, Caroline O. Michie, Tzyvia Rye, Michael Churchman, Amelia Hallas-Potts, Ian Croy, W. Glenn McCluggage, Alistair R.W. Williams, Clare Bartos, Yasushi Iida, Aikou Okamoto, Brian Dougherty, J. Carl Barrett, Ruth March, Athena Matakidou, Patricia Roxburgh, Colin A. Semple, D. Paul Harkin, Richard Kennedy, C. Simon Herrington, Charlie Gourley

**Affiliations:** 1 Nicola Murray Centre for Ovarian Cancer Research, Cancer Research UK Scotland Centre, Institute of Genetics and Cancer, University of Edinburgh, Edinburgh, UK.; 2 MRC Human Genetics Unit, Institute of Genetics and Cancer, University of Edinburgh, Edinburgh, UK.; 3 Edinburgh Cancer Centre, Western General Hospital, NHS Lothian, Edinburgh, UK.; 4 Department of Pathology, Belfast Health and Social Care Trust, Belfast, UK.; 5 Division of Pathology, The Royal Infirmary of Edinburgh, Edinburgh, UK.; 6 The Jikei University School of Medicine, Tokyo, Japan.; 7 Translational Medicine, Oncology R&D, AstraZeneca, Waltham, Massachusetts.; 8 Precision Medicine and Biosamples, Oncology R&D, AstraZeneca, Cambridge, UK.; 9 Centre for Genomics Research, Discovery Sciences, BioPharmaceuticals R&D, AstraZeneca, Cambridge, UK.; 10 Institute of Cancer Sciences, Wolfson Wohl Cancer Research Centre, University of Glasgow, Belfast, UK.; 11 Beatson West of Scotland Cancer Centre, Glasgow, UK.; 12 Almac Diagnostics, Craigavon, UK.; 13 Centre for Cancer Research and Cell Biology, Queen's University Belfast, Belfast, UK.

## Abstract

**Purpose:**

High-grade serous ovarian carcinoma (HGSOC) is the most common ovarian cancer type; most patients experience disease recurrence that accumulates chemoresistance, leading to treatment failure. Genomic and transcriptomic features have been associated with differential outcome and treatment response. However, the relationship between events at the gene sequence, copy number, and gene-expression levels remains poorly defined.

**Experimental Design:**

We perform multiomic characterization of a large HGSOC cohort (*n* = 362) with detailed clinical annotation to interrogate the relationship between patient subgroups defined by specific molecular events.

**Results:**

*BRCA2*-mutant (*BRCA2*m) and *EMSY*-overexpressing cases demonstrated prolonged survival [multivariable hazard ratios (HR) 0.40 and 0.51] and significantly higher first- and second-line chemotherapy response rate. *CCNE1*-gained (*CCNE1*g) cases demonstrated underrepresentation of FIGO stage IV cases, with shorter survival but no significant difference in treatment response. We demonstrate marked overlap between the TCGA- and Tothill-derived subtypes. IMR/C2 cases displayed higher *BRCA1/2*m frequency (25.5%, 32.5%) and significantly greater immune cell infiltration, whereas PRO/C5 cases had the highest *CCNE1*g rate (23.9%, 22.2%) and were uniformly low in immune cell infiltration. The survival benefit for cases with aberrations in homologous recombination repair (HRR) genes was apparent across all transcriptomic subtypes (HR range, 0.48–0.68). There was significant co-occurrence of RB loss and HRR gene aberrations; RB loss was further associated with favorable survival within HRR-aberrant cases (multivariable HR, 0.50).

**Conclusions:**

These data paint a high-resolution picture of the molecular landscape in HGSOC, better defining patients who may benefit most from specific molecular therapeutics and highlighting those for whom novel treatment strategies are needed to improve outcomes.

**Translational Relevance**

Molecular features of high-grade serous ovarian carcinoma have been associated with outcome and treatment response. Understanding associations between these different features is important for identifying optimal management strategies for patients. However, the relationship between molecular events at the gene sequence, copy number, and gene-expression levels is poorly understood. We explore these associations and the clinical impact of specific molecular events in a large patient cohort, highlighting patient subgroups with differential survival and therapy sensitivity. We identify significant relationships between subgroups defined by gene-expression patterns, mutation events, copy number events, and levels of infiltrating immune cells. These data suggest specific therapies—such as PARP inhibitors, agents targeting the cell cycle, and immunotherapies—are most likely to benefit particular patient populations. Moreover, loss of RB expression, which frequently occurs alongside homologous recombination defects, may affect patient survival in this context.

## Introduction

High-grade serous ovarian carcinoma (HGSOC) is the most common form of tubo-ovarian cancer. The majority of patients with HGSOC are diagnosed at advanced stage and experience poor prognosis, with a five-year survival of approximately 30% in this population (1). Although the majority of HGSOC demonstrate high levels of intrinsic sensitivity to platinum-based chemotherapy, most patients experience disease recurrence that accumulates therapy resistance, leading to progressively shorter treatment-free intervals until patients eventually succumb to the disease (2, 3).

In the hope of identifying therapeutically exploitable disease biology, a wealth of data have been produced over the last two decades characterizing the genomic and transcriptomic landscape of HGSOC (4–7). At the gene sequence level, the identification of mutational disruption in *BRCA1* and *BRCA2* (*BRCA1*/*2*m) has ultimately paved the way for the integration of poly-(ADP-ribose) polymerase (PARP) inhibitor use into routine care for some patients (8–10). Indeed, there continues to be an intense research effort surrounding mechanisms and implications of homologous recombination DNA repair (HRR) disruption beyond *BRCA1*/*2*m (11); these include mutation of non-*BRCA1*/*2* HRR genes (12), large-scale genomic variants disrupting *BRCA1*/*2* (13), epigenetic inactivation of HRR players such as *BRCA1* and *RAD51C* (5, 14), and overexpression of the BRCA2 regulator EMSY (15, 16).

At the gene-expression level, numerous studies have characterized HGSOC samples, endeavoring to identify clinically meaningful transcriptomic subtypes of disease or expression signatures predictive of survival risk (4, 5, 7, 17, 18). Most notably, Tothill and colleagues (4) and the TCGA investigators (5) each identified multiple transcriptomic subtypes, associating these with differential survival profiles. These analyses have identified favorable outcomes in patients with tumors harboring expression profiles suggestive of active immune engagement (TCGA IMR subtype, refs. 5, 19; Tothill C2 subtype, ref. 4), consistent with earlier reports of favorable outcomes in cases with high levels of cytotoxic T-cell infiltration (20, 21). However, transcriptomic subtyping is not currently used for clinical prognostication or stratification of patients with HGSOC, despite some investigators reporting the differential sensitivity of these groups to agents such as bevacizumab (22).

Although multiple investigators have characterized either the genomic or transcriptomic landscape of HGSOC, few have investigated the relationship between genomic and transcriptomic features. Moreover, the relationship between these events and recently identified recurrent disruption of RB and PTEN in HGSOC is poorly understood (6). Integration of multiple layers of molecular characterization is required to paint a granular picture of the molecular landscape in HGSOC to better inform rationally designed trials of novel treatment regimens or combination therapy strategies. Indeed, some investigators have suggested that transcriptomic subtypes of HGSOC that appear to derive the greatest benefit from antiangiogenic agents may be depleted for *BRCA1*/*2*m cases that benefit most from PARP inhibition (5, 22). This notion is consistent with mixed results observed from the addition of antiangiogenic agents to PARP inhibitors (PARPi) dependent on patient selection, therapy line, and agent combinations (23), exemplifying the need for comprehensive multilayer molecular characterization to inform patient selection for future investigations of novel treatment approaches.

Here we perform matched the genomic and transcriptomic characterization of a large, well-annotated HGSOC cohort, dissecting the relationship between patient groups defined at the gene sequence, gene copy number, and gene-expression levels.

## Materials and Methods

### Patient cohort

Five hundred thirty-nine patients with ovarian cancer treated at the Edinburgh Cancer Centre met the following study inclusion criteria: primary ovarian, peritoneal, or fallopian tube carcinoma (any histologic type) diagnosed prior to 2007; available formalin-fixed paraffin-embedded (FFPE) treatment-naïve surgical specimen; first-line platinum-containing chemotherapy; minimum 3-year follow-up. Pathology review of H&E-stained slides was undertaken by expert gynecological pathologists (W.G. McCluggage, A.R.W. Williams, and C.S. Herrington) to identify HGSOC cases (Supplementary Fig. S1); IHC for p53 and WT1 was used to clarify cases of uncertain histologic type (HGSOC: WT1 positive, p53 aberrant mutation-type expression pattern; Supplementary Methods Section 1; Supplementary Fig. S1). Ethical approval was obtained from South East Scotland Human Annotated Bioresource (Lothian NRS Bioresource Ethics Committee reference 15/ES/0094-SR705 and SR752). The need for consent was waived by the ethics committee due to the retrospective nature of the study. This study was carried out in accordance with the principles of the Declaration of Helsinki.

### Genomic characterization

H&E-stained slides were marked to identify tumor areas of high cellularity and used as a guide for macrodissection of 10-μm FFPE sections for DNA extraction. DNA extraction was performed using the QIAamp FFPE DNA Kit and Qiagen Deparaffinization Solution (Qiagen). Extracted DNA was quantified by high sensitivity Qubit assay. *CCNE1* and *EMSY* copy number (CN) was quantified by TaqMan qPCR (Supplementary Methods Section 2). Samples with ≥4 copies were considered gained for *CCNE1*; samples with ≥6 copies of *EMSY* were considered amplified.

High-throughput sequencing was performed using a custom Integrated DNA Technologies gene capture panel with unique molecular indices (Supplementary Methods Section 3). Whole-genome libraries were generated, pooled for target capture (see Supplementary Methods Section 3 for a full list of target genes designed around known HGSOC driver events and homologous recombination repair-associated genes), and sequenced using an Illumina NextSeq 550 at the Edinburgh Clinical Research Facility, Western General Hospital, Edinburgh, UK. The median per-sample mean target coverage was 593× (range, 205–3,278×). Reads were processed using the bcbio v1.0.6 high-throughput sequence analysis pipeline (Supplementary Methods Section 4). Consensus reads aligned to hg38 underwent variant calling using a majority vote system from three variant callers (Freebayes, VarDict, and Mutect2). Called variants were annotated using the Ensembl VEP v90.9 against Ensembl release 90 and filtered to retain only functional variation (Supplementary Methods Section 5).

### Transcriptomic subtyping

Transcriptomic data for the cohort were available from previous work identifying transcriptomic subtypes of HGSOC (15, 17), including *EMSY*-overexpression status (Supplementary Methods Section 6). Briefly, samples from otherwise unselected patients who received adjuvant platinum-based chemotherapy were characterized in this previous work as a training (*n* = 247 HGSOC) and validation (*n* = 115 HGSOC) cohort. RNA was extracted from macrodissected FFPE, cDNA was amplified, fragmented, and labeled prior to hybridization to the Ovarian DSA cDNA microarray platform. Raw transcriptomic data were normalized using the Robust Multi-Array Average method prior to a quality control. TCGA (MES, PRO, IMR, and DIF) and Tothill (C1, C2, C4, and C5) transcriptomic subtyping calls were made with the consensusOv R package using the consesusOv and Helland approaches (ref. 19; Supplementary Methods Section 6).

### Immune cell infiltration analysis

Tumor-infiltrating CD3-positive and CD8-positive immune cells were quantified by IHC of constructed tumor tissue microarrays (TMA; Supplementary Methods Section 7); marker-positive cell burden was quantified as percentage positive cells within tumor areas using QuPath version 0.1.2 (24).

### Detection of PTEN and RB loss by IHC

PTEN and RB protein loss was detected by IHC using sections of the HGSOC TMA (Supplementary Methods Section 8). Loss was defined as a complete absence of positive staining in tumor cells with confirmed corresponding positive internal control stromal staining.

### CN analysis from off-target sequencing reads

CN analysis was performed using CopywriteR (ref. 25; Supplementary Methods Section 9): off-target reads were used to estimate the relative CN of 50 kB genome segments across each chromosome, using the alignment bam files from the above sequencing analysis workflow. For quantification of CN alteration burden, adjacent 50 kB segments of gain/loss representing the same large CN event were merged prior to quantification (Supplementary Methods Section 9).

### Clinical annotation

Baseline clinicopathologic features and outcome data were extracted from the Edinburgh Ovarian Cancer Database (1), alongside chemotherapy response data (Supplementary Methods Section 10). Overall survival (OS) and progression-free survival (PFS) were defined as the time from pathologically confirmed diagnosis to patient death and disease progression or recurrence, respectively (Supplementary Methods Section 9).

### Statistical analyses

All statistical analyses were performed using R version 4.0.3 (R Foundation for Statistical Computing, Vienna, Austria). Comparisons of frequency were performed using the Chi-squared test or Fisher exact test, as appropriate. Between-group comparisons of continuous variables were performed using the Mann–Whitney *U* test. Survival analysis was performed using Cox proportional hazards regression models and reported as hazard ratios (HR) with 95% confidence intervals (95% CI). For survival analysis adjusted for other clinicopathologic factors, multivariable hazard ratios (mHR) are reported. Median follow-up time was calculated by the reverse Kaplan–Meier method. Adjustment for multiple testing was applied using the Bonferroni method.

### Data availability

All data are available upon reasonable request to the corresponding author, subject to requests falling within our local ethics framework. Normalized gene-level transcriptomic data are included as an appendix to this manuscript.

## Results

### Cohort characteristics

Of the 539 ovarian cancer cases that met eligibility criteria, 362 were classified as HGSOC following pathology review and underwent molecular characterization (*n* = 27 insufficient tumor, *n* = 141 non-HGSOC, *n* = 1 failed sequencing library preparation, *n* = 8 failed quality control; Supplementary Fig. S1). Clinicopathologic features of the study cohort are summarized in Table 1. The median follow-up time was 15.0 years.

**Table 1.** Clinicopathologic features of patients with HGSOC.

		*N*	%
Total cases	*N*	362	
Age at diagnosis	Median years	61	Range, 33–86
FIGO stage at diagnosis	I	15	4.3
	II	31	8.8
	III	237	67.5
	IV	68	19.4
	NA	11	–
RD following surgical debulking	No visible RD (0 cm)	65	19.4
	Macro RD (0.1–2 cm)	66	19.7
	Gross macro RD (≥2 cm)	187	55.8
	Macro RD of unknown size	17	5.1
	Unknown	27	–
First-line chemotherapy	Single-agent platinum	217	59.9
	Platinum–taxane combination	135	37.3
	Other platinum-containing regimes	10	2.8
Vital status at last follow-up	Alive	36	9.9
	Deceased—died of OC	307	84.8
	Deceased—other causes	19	5.2
Median follow-up time	Years	15.0 (95% CI, 12.8–19.9)	
Median PFS	Years	1.17 (95% CI, 1.09–1.28)	
Median OS	Years	2.60 (95% CI, 2.40–3.07)	

Abbreviations: FIGO, International Federation of Gynecology and Obstetrics; NA, not available; Macro, macroscopic; RD, maximal residual disease diameter; OC, ovarian carcinoma.

### Molecular landscape of HGSOC

**Figure 1.**
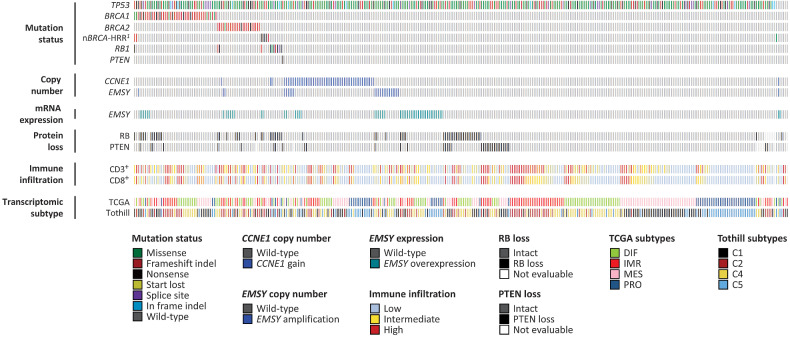
Molecular landscape of HGSOC. ^1^Mutation in non-*BRCA1*/*2* HRR genes: 3 *BRIP1*, 2 *CHEK2*, 1 *RAD51C*, 1 *PALB2*, 1 concurrent *BAP1* and *NBN*. *CCNE1* CN gain, ≥4 copies by TaqMan CN assay. *EMSY* CN amplification, ≥6 copies by TaqMan CN assay.

The frequency of *TP53* mutation was 98.1% (355 of 362 cases; Fig. 1; Supplementary Table S1). 12.7% and 6.6% of cases harbored *BRCA1*m and *BRCA2*m. Eight cases (2.2%) demonstrated mutation of other HRR genes (3 *BRIP1*, 2 *CHEK2*, 1 *RAD51C*, 1 *PALB2*, 1 concurrent *BAP1* and *NBN*). 14.9% of cases displayed CN gain of *CCNE1* (*CCNE1*g) and 6.6% demonstrated amplification of *EMSY*. Tumors demonstrating *EMSY* amplification were enriched for *EMSY* mRNA-overexpressing cases (*P* < 0.001; Supplementary Table S2); however, *EMSY* CN was a poor predictor of *EMSY*-overexpression status (positive predictive value 0.42; 95% CI, 0.22–0.63; negative predictive value, 0.88; 95% CI, 0.84–0.91).

**Figure 2.**
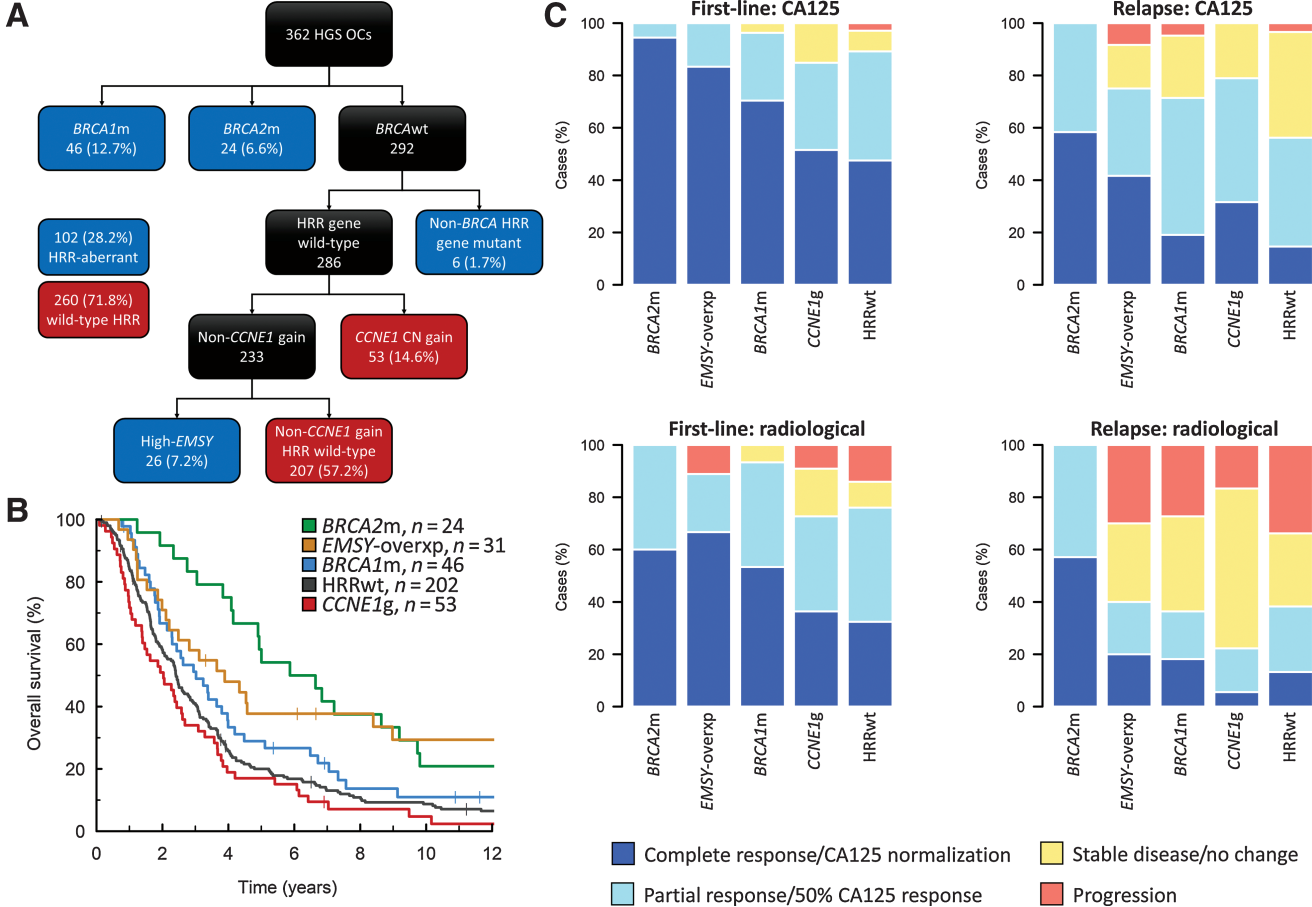
HRR pathway (HRR)–centric subtyping of high-grade serous carcinoma. **A,** HRR-centric classification taxonomy. **B,** OS profile of HRR-centric subtypes. **C,** Chemosensitivity of HRR-centric subtypes at first-line treatment (left) and treatment for disease relapse (right) as determined by CA125 tumor marker (top) and radiology (bottom). *BRCA2*m, *BRCA2* mutant; *BRCA1*m, *BRCA1* mutant; *EMSY*-overxp; overexpression of *EMSY*; *CCNE1*g, gain of *CCNE1*; HRRwt, non-*CCNE1*g HRR wild-type.

An HRR-centric stepwise taxonomy was constructed (Fig. 2A). Compared with the non-*CCNE1*g HRR-wild-type reference population (HRRwt; 55.8% of cases), *BRCA2*m and *EMSY*-overexpressing (8.6%) cases demonstrated favorable outcome (mHR for OS = 0.40; 95% CI, 0.25–0.64 and 0.51; 95% CI, 0.32–0.81; Fig. 2B; Supplementary Fig. S2; Supplementary Table S3). Stage IV cases were underrepresented in the *CCNE1*g group (8.2% vs. 23.4% in the HRRwt group, *P* = 0.017); *CCNE1*g cases demonstrated significantly shorter survival after accounting for age, stage, and debulking status (mHR for OS = 1.52; 95% CI, 1.11–2.09). The *BRCA2*m and *EMSY*-overexpressing subgroups demonstrated the highest rates of complete response to first-line chemotherapy (Fig. 2C); these groups also demonstrated the highest rates of complete response to chemotherapy at first relapse. The complete response rate was higher in the *BRCA2*m, *EMSY*-overexpressing and *BRCA1m* cases compared with the HRRwt cases at first chemotherapy [*P* < 0.001, *P* = 0.009 and *P* = 0.049 for complete GCIG CA125 response (confirmed normalization from at least double upper limit of normal)]; however, only *BRCA2*m and *EMSY*-overexpressing cases had a significantly higher complete response rate after adjusting for multiple testing (Bonferroni-adjusted *P* = 0.001, 0.027, and 0.148, respectively). At relapse, *BRCA2*m and *EMSY*-overexpressing cases retained a higher chemotherapy response rate (*P* = 0.002 and *P* = 0.037 for complete CA125 response, respectively). The chemotherapy response rate was similar in the *CCNE1*g and HRRwt groups at both primary treatment and relapse (Fig. 2C).

### Relationship between transcriptomic subtypes

**Figure 3.**
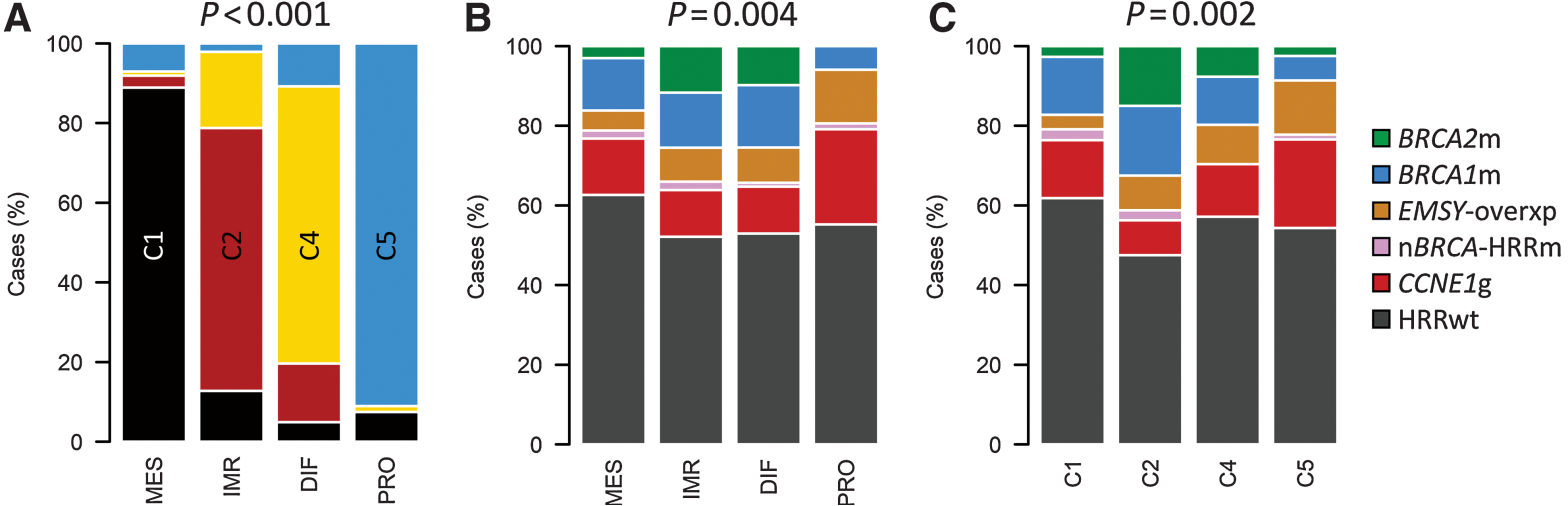
Relationship between subtyping methodologies. **A,** Comparison of transcriptomic subgrouping approaches: composition of Tothill subtypes across each of the TCGA subtypes; labeled *P* value represents comparison of Tothill subtype frequency across all TCGA subtypes by the Chi-squared test. **B,** Distribution of homologous recombination repair (HRR)-centric subtypes across each of the TCGA transcriptomic subtypes; labeled *P* value represents comparison of *BRCA1*/*2*m frequency across all groups by Chi-squared test; Bonferroni-adjusted *P* = 0.009. **C,** Distribution of HRR-centric subtypes across each of the Tothill transcriptomic subtypes; labeled *P* value represents comparison of *BRCA1*/*2*m frequency across all groups by the Chi-squared test; Bonferroni-adjusted *P* = 0.003. *BRCA2*m, *BRCA2* mutant; *BRCA1*m, *BRCA1* mutant; *EMSY*-overxp; overexpression of *EMSY*; nBRCA-HRRm, non-*BRCA1*/*2* HRR gene mutation; *CCNE1*g, gain of *CCNE1*; HRRwt, non-*CCNE1*g HRR wild-type.

Two transcriptomic subtyping approaches were used (TCGA subtypes: DIF, IMR, PRO, and MES; Tothill subtypes: C1, C2, C4, and C5). There was marked overlap between the subtyping approaches (*P* < 0.0001; Fig. 3A; Supplementary Table S4): PRO cases were overwhelmingly of the C5 subtype (91.0%, 61 of 67; Supplementary Table S4), whereas the vast majority of MES cases were of the C1 subtype (88.9%, 88 of 99). The DIF group comprised mainly C4 tumors (69.6%, 71 of 102), whereas IMR cases were mostly of the C2 subtype (66.0%, 62 of 94).

### Genomic–transcriptomic correlates

There was a marked association between HRR-centric and transcriptomic subtypes (Fig. 3B and C). The frequency of *BRCA1* and *BRCA2* mutation differed significantly between transcriptomic subtypes, with the highest and lowest *BRCA1*/*2*m rates in the IMR/C2 and PRO/C5 subtypes, respectively (25.5% and 32.5% vs. 6.0% and 8.6%, Bonferroni-adjusted *P* = 0.009 and 0.003; Fig. 3B and C). The frequency of *CCNE1g* was highest in PRO/C5 tumors (23.9% in PRO, 22.2% in C5), whereas the C2 subtype demonstrated the lowest *CCNE1*g frequency (8.8%, *P* = 0.002; Fig. 3B and C).

Prolonged survival for cases with HRR gene aberrations (*BRCA1*m, *BRCA2*m, *EMSY*-overexpression or non-*BRCA*-HRR mutation) was apparent across all transcriptomic subtypes (HR range, 0.48–0.68; Supplementary Figs. S3 and S4). We did not observe any significant differences in the overall burden of CN loss or gain events between transcriptional subtypes (Supplementary Fig. S5).

### Immune cell infiltration burden

**Figure 4.**
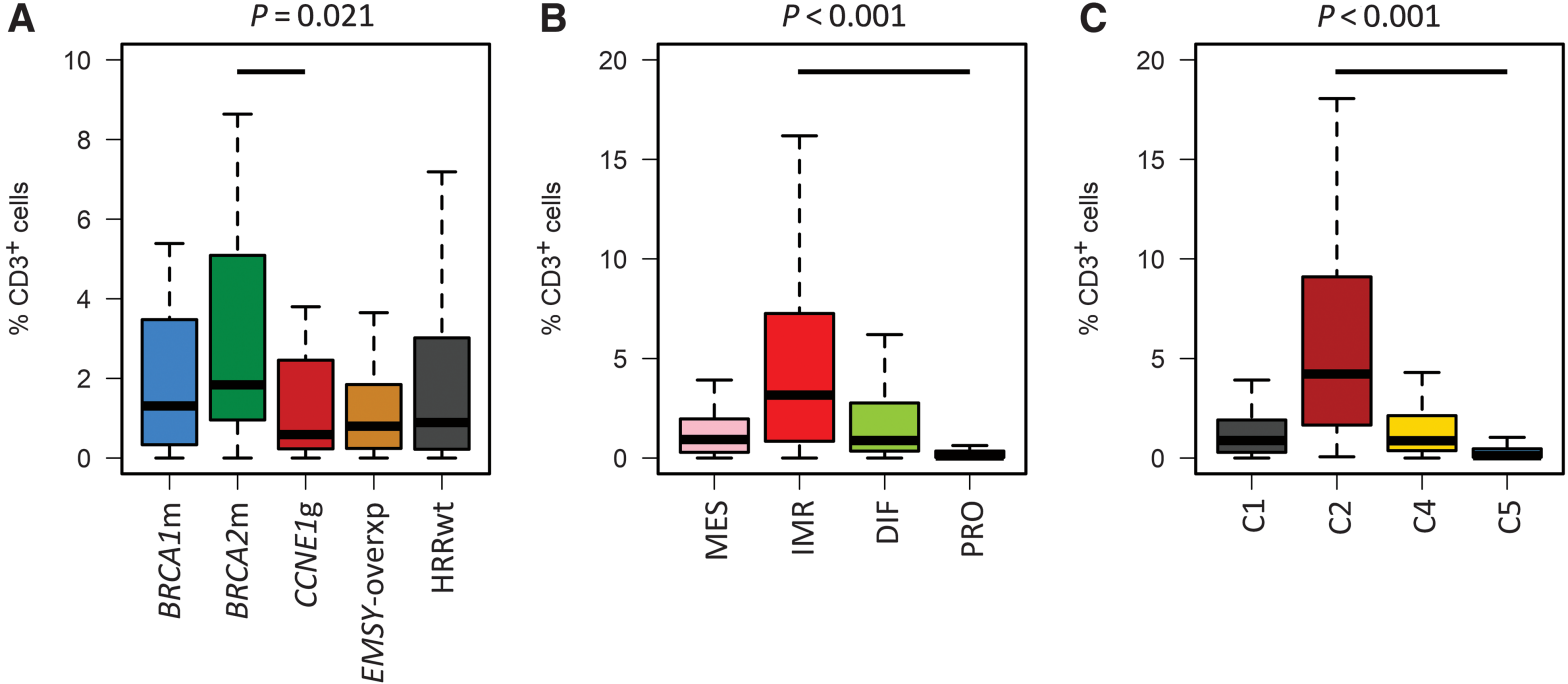
Tumor-infiltrating immune cells across high-grade serous carcinoma subtypes. **A,** CD3^+^ infiltration across HRR-centric subtypes; labeled *P* value represents comparison of *BRCA2*m and *CCNE1*g groups using the Mann–Whitney *U* test. **B,** CD3^+^ infiltration across TCGA transcriptomic subtypes; labeled *P* value represents comparison of IMR and PRO groups using the Mann–Whitney *U* test. **C,** CD3^+^ infiltration across Tothill transcriptomic subtypes; labeled *P* value represents comparison of C2 and C5 groups using the Mann–Whitney *U* test. *BRCA2*m, *BRCA2* mutant; *BRCA1*m, *BRCA1* mutant; *EMSY*-overxp; overexpression of *EMSY*; *CCNE1*g, gain of *CCNE1*; HRRwt, non-*CCNE1*g HRR wild-type.

The burden of tumor-infiltrating CD3^+^ and CD8^+^ cells was heterogeneous across samples, with higher infiltration associated with prolonged survival (Supplementary Fig. S6). *BRCA2*m cases demonstrated the highest levels of CD3^+^ infiltration (Fig. 4A).

Subtypes defined by both transcriptomic subgrouping methodologies demonstrated marked differences in infiltrating CD3^+^ (Bonferroni-adjusted *P* < 0.0001; Fig. 4B and C) and CD8^+^ cells (Bonferroni-adjusted *P* < 0.0001; Supplementary Fig. S7). The IMR/C2 subtypes demonstrated the highest infiltration levels, whereas the PRO/C5 subtypes demonstrated uniformly low levels of infiltrating cells.

### RB and PTEN loss in HGSOC

**Figure 5.**
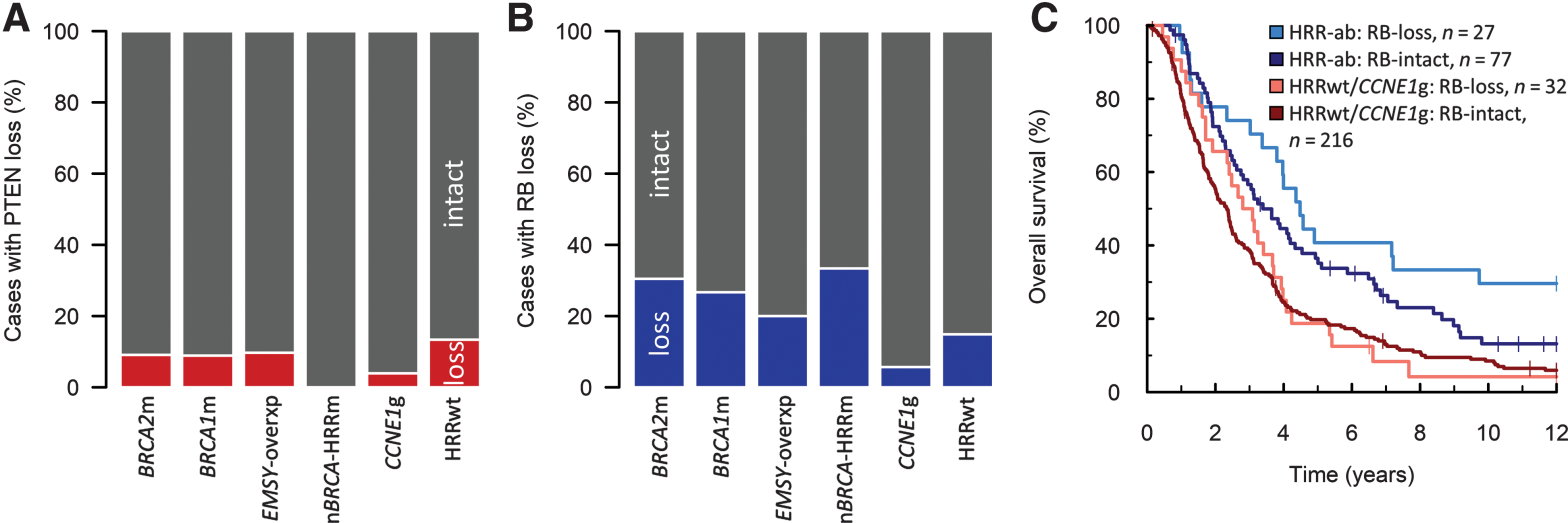
PTEN and RB loss in HGSOC. **A,** Frequency of loss of PTEN protein expression across HRR-centric subtypes. **B,** Frequency of loss of RB protein expression across HRR-centric subtypes. **C,** Impact of RB loss on survival in patients based on HRR status. Multivariable hazard ratio (mHR) for HRR-ab: RB loss vs. HRR-ab RB-intact = 0.50; 95% CI, 0.30–0.84; mHR for HRRwt/*CCNE1*g: RB loss vs. HRRwt/*CCNE1*g: RB-intact = 0.71; 95% CI, 0.48–1.06. *BRCA2*m, *BRCA2* mutant; *BRCA1*m, *BRCA1* mutant; *EMSY*-overxp; overexpression of *EMSY*; nBRCA-HRRm, non-*BRCA1*/*2* HRR gene mutation; *CCNE1*g, gain of *CCNE1*; HRRwt, non-*CCNE1*g HRR wild-type. HRR-ab, HRR-aberrant: *BRCA1*m, *BRCA2*m, *EMSY*-overxp, or n*BRCA*-HRRm.

10.6% of cases (37 of 350 evaluable tumors) demonstrated PTEN protein loss (Fig. 1; Fig. 5A). PTEN loss was a rare event in tumors of the PRO/C5 subtypes (3.0% in PRO, 2.5% in C5; Supplementary Fig. S8A and S8B). Cases with loss of PTEN expression demonstrated significantly lower *PTEN* CN (*P* = 0.0003; Supplementary Fig. S9A).

16.8% of cases (59 of 352 evaluable tumors) demonstrated loss of RB protein (Fig. 1; Supplementary Fig. S8C and S8D). RB loss was ubiquitous among HGSOCs harboring *RB1* mutation (11/11 cases demonstrating loss; *P* < 0.001 vs. 48/341 in the absence of *RB1* mutation; Fig. 1). Cases demonstrating RB loss had a lower *RB1* CN (*P* = 0.0258; Supplementary Fig. S9B), and there was significant co-occurrence between RB loss and PTEN loss (22.8% PTEN loss in RB-lost cases, 13/57 vs. 7.7%, 22/285 evaluable cases; *P* = 0.001; Fig. 1).

RB loss was significantly enriched among cases with HRR gene aberrations (26.0%, 27/104 evaluable cases vs. 12.9%, 32/248; *P* = 0.005; Fig. 5B) and was a rare event among *CCNE1*g cases (5.7%). In cases with HRR gene aberrations, RB loss was associated with significantly longer survival (mHR for OS = 0.50; 95% CI, 0.30–0.84; Fig. 5C); conversely, RB loss was not associated with significant differences in survival within the remaining population (mHR = 0.71; 95% CI, 0.53–1.06; Fig. 5C).

### Molecular profile of long-term survivors of late-stage HGSOC

Fifty-three patients were alive 5 years following diagnosis of advanced-stage (FIGO III/IV) HGSOC. Ten cases (18.9%) were *BRCA2*m and 8 were *BRCA1*m (15.1%); 6 cases were in the *EMSY*-overexpression HRR-centric subtype (11.3%), and 1 case was in the non-*BRCA* HRR gene mutant group (1.9%). Three cases had *CCNE1*g (5.7%), and the remaining 25 were in the HRRwt group (47.2%). Ten percent of cases had PTEN loss (5 of 49 evaluable cases) and 29.4% had RB loss (15 of 51 evaluable cases). These cases demonstrated a high tumor-infiltrating immune cell burden (median percentage CD3- and CD8-positive cells 1.8% and 1.1%, versus 0.9% and 0.5% across the whole cohort).

### Association of molecular features with complete resection

Molecularly defined subgroups were interrogated for association with rates of complete resection. HRR-aberrant cases (*BRCA1*/*2*m, *EMSY* overexpression, and non-*BRCA* HRR gene mutation) demonstrated a higher rate of complete debulking compared with HRRwt cases, but this did not cross the threshold for statistical significance after adjusting for multiple testing (Bonferroni-adjusted *P* = 0.066; Supplementary Table S5). The IMR transcriptomic subtype was associated with significantly higher rates of complete resection (Bonferroni-adjusted *P* = 0.0134), and complete resection rates were also significantly higher in cases with high CD8^+^ infiltration (Bonferroni-adjusted *P* = 0.007; Supplementary Table S5).

## Discussion

Substantial advancements in our understanding of HGSOC biology have been made over the last two decades, with many studies characterizing HGSOC cases at the gene sequence or gene-expression level (4–6, 19). These investigations have identified subgroups of patients with differential outcome and therapy sensitivity, paving the way for molecular stratification of HGSOC patient care (8, 22, 26). However, the relationship between features described at the genomic and transcriptomic level is poorly understood. We present matched genomic–transcriptomic characterization—alongside identification of other molecular features, including RB expression loss, PTEN expression loss, and immune cell infiltration—in a large pathologically confirmed HGSOC cohort with detailed clinical annotation and extensive follow-up (median 15 years), revealing marked correlation across these levels of molecular characterization.

We utilized two transcriptomic subtyping approaches within our data set: There was a substantial correlation between TCGA (PRO, MES, DIF, and IMR) and Tothill (C1, C2, C4, and C5) subtypes. The MES and PRO TCGA subtypes demonstrated marker overlap with the C1 and C5 Tothill subtypes, whereas the majority of DIF and IMR cases were of the C4 and C2 subtypes, respectively. This overlap is consistent with previous reports of overlap between these subtyping approaches (19).

When comparing genomic features of these subtypes, the IMR/C2 groups demonstrated enrichment for *BRCA1*/*2*m. These cases also demonstrated the highest immune cell infiltration burden, with significantly greater levels of CD3^+^ and CD8^+^ cell infiltration. Together, the high *BRCA1*/*2*m rate and high levels of immune engagement in IMR/C2 tumors likely underpin the favorable outcome reported in these patient groups (17, 19). In contrast, the vast majority of PRO/C5 cases were *BRCA1*/*2* wild-type and instead demonstrated the highest rates of *CCNE1*g; the PRO/C5 subtypes may therefore represent the group least likely to benefit from PARPi. Previous reports have suggested that PRO cases may derive the greatest benefit from antiangiogenic agents such as bevacizumab (22); the low *BRCA1*/*2*m rate in this group may support the use of these agents over PARPi in this patient group. Conversely, the IMR/C2 group harbors a large number of *BRCA1*/*2*m patients who are likely to benefit from PARPi. Some investigators have suggested that antiangiogenic therapies may not confer greatest benefit in patients with HGSOC demonstrating immune-related gene-expression signatures (17) or may not benefit some groups of HRR-deficient patients (15, 27, 28). This includes a recent large retrospective case-controlled analysis of bevacizumab-treated patients who suggested the PFS benefit for bevacizumab was limited to the *BRCA1*/*2* wild-type population (27), and translational analysis of GOG-218 demonstrating no significantly improved PFS in cases with HRR gene mutations (28). However, the positive findings from the PAOLA-1 trial combining olaparib with bevacizumab suggest that, whereas HRR-deficient patients may not derive the greatest benefit from bevacizumab alone, the combination of bevacizumab and PARPi is clearly efficacious within this population (29); unfortunately, PAOLA-1 did not include an olaparib-only treatment arm for direct comparison of olaparib versus olaparib-bevacizumab maintenance. Together, these data suggest that further dissection of the relationship between transcriptional subtypes, HRR status, and relative benefit of single versus combined PARPi/antiangiogenic strategies is required.

PRO/C5 cases were uniformly low in tumor-infiltrating CD3^+^ and CD8^+^ cells, suggesting poor engagement of the immune system against the tumor within this patient group; this may partially account for the shorter survival time previously described in these cases (17, 19). These data suggest that immune-checkpoint inhibitors, currently under investigation in ovarian cancer, may not improve outcomes in these patients without additional interventions that affect the tumor microenvironment. Although the expression of immunosuppressive molecules has been identified in the HGSOC tumor microenvironment, response rates to immune-checkpoint inhibitors have been modest in the context of ovarian cancer (around 10% in the KEYNOTE-100 trial of pembrolizumab monotherapy; ref. 30). Combinations of checkpoint inhibitors with PARPi have become of great interest, with the TOPACIO/KEYNOTE-162 trial of pembrolizumab and niraparib showing efficacy in platinum-resistant patients regardless of *BRCA1*/*2* status (objective response rate 18%; ref. 31); a later analysis identified HRR deficiency-related mutational signature 3 and exhausted CD8^+^ T cells as markers of response. Lampert and colleagues performed biomarker analysis of tumors from patients in a phase II study of olaparib and the PD-L1 inhibitor durvalumab, suggesting that HGSOC patients of the IMR subtype may be most likely to benefit (32). Together, these data suggest that the comprehensive molecular profiling of cases in such trials may help determine which patients are mostly likely to benefit from immune-checkpoint blockade with or without PARPi; this characterization should include identification of key transcriptomic subtypes, immune cell profiling, and assessment of *BRCA1*/*2*, HRR, and *CCNE1*g status.

Our multilayer characterization sheds further light upon HRR pathway players and their importance in HGSOC. We show that *EMSY*-overexpressing cases appear *BRCA2*m-like in their survival profile and therapy sensitivity—consistent with EMSY's role as a BRCA2 regulator (16). However, they do not appear to be overrepresented in the IMR/C2 transcriptomic subtypes and do not demonstrate a higher burden of tumor-infiltrating immune cells. We also demonstrate the importance of aberrations in HRR genes regardless of the transcriptional subtype context; the hazard ratio for cases with HRR gene aberrations ranged between 0.48 and 0.68 across all transcriptional subtypes. These data confirm that the survival benefit among patients with tumors displaying HRR gene aberrations is not due to differential distribution of transcriptional subtypes.

*CCNE1*g has been the focus of intense research interest since its identification as a recurrent event in HGSOC (5, 33–35). A number of studies have suggested that cases harboring *CCNE1*g have poorer survival, with some suggesting this is due to greater intrinsic chemoresistance (5, 33–35). However, these comparisons have typically been made against the wider non-*CCNE1*g population without accounting for HRR deficiency, which is associated with longer survival and increased platinum sensitivity, confounding these comparisons. We compare the *CCNE1*g population directly to non-*CCNE1*g HHRwt cases. *CCNE1*g was not associated with a significantly poorer response rate to first-line chemotherapy or chemotherapy for relapsed disease, within our cohort. We show that although the most advanced-stage cases are underrepresented in the *CCNE1*g group, *CCNE1*g cases demonstrate shorter survival time and that their survival is significantly poorer compared with non-*CCNE1*g HRRwt patients upon multivariable analysis. *CCNE1*g tumors also demonstrated the lowest levels of infiltrating immune cells compared with the other HRR-centric groups, which may contribute toward the shorter patient survival time. Mutual exclusivity of *CCNE1*g and HRR gene mutations suggests that the former are likely to represent a patient group who benefit least from PARP inhibition. Moreover, the low immune infiltration levels demonstrated by these cases suggest that immune-checkpoint inhibitors may not be effective monotherapies in these patients (36). Given that *CCNE1g* is most frequent in the PRO/C5 transcriptomic subtypes (22), and that the PRO subtype has been associated with greatest benefit from bevacizumab in some reports, *CCNE1*g cases may represent those likely to derive benefit from antiangiogenic therapies.

*CCNE1*g cases—alongside other HRR-proficient patient groups—represent patients with HGSOC with shorter survival time for which new treatment approaches are needed to improve survival. However, the low frequency of other molecular events in *CCNE1*g cases (*BRCA1*/*2* wild-type, RB-intact, PTEN intact, low immune cell infiltration) represents a challenge toward identifying further candidate biologically targeted strategies within this patient group. Cyclin E1, the gene product of *CCNE1*, complexes with CDK2 to drive cell-cycle progression from G_1_ into S phase (37); novel agents targeting the cell cycle have therefore become attractive as potential therapeutic options for *CCNE1*g HGSOC. Inhibitors of WEE1—a regulator of G_1_–S and G_2_–M entry (38)—represents one such therapy, with inhibition leading to premature cell-cycle progression, increased replication stress and mitotic catastrophe. Recent data have demonstrated objective responses to WEE1 inhibitors in treatment-refractory *CCNE1*g HGSOC (39). Inhibition of ATR, which regulates G_2_–M progression in response to replication stress (38), is also of interest in this patient group, and a recent study demonstrated the synergistic effect of combining ATR and WEE1 inhibitors in *CCNE1*g patient-derived xenograft models (40). Assessment of *CCNE1*g status in patients enrolled in trials of cell cycle–directed therapies is warranted to identify whether these represent a feasible strategy specifically for this poor prognosis group.

Disruption of *PTEN* and *RB1* has only recently been identified as highly recurrent events in HGSOC (6). The relationship of these events to other molecular features and their impact on patient outcomes are poorly understood. We identified PTEN loss in 10.6% of our cases. This frequency is lower than that reported by the OTTA consortium (18.9%; ref. 41); however, not all centers in the OTTA study performed pathology review of histologic slides (42), and it is, therefore, feasible that this may be an overestimation due to inclusion of a minority of pseudo-serous endometrioid ovarian carcinomas, which display a high frequency of PTEN disruption (41, 43). We demonstrate that PTEN and RB protein loss is neither mutually exclusive with one another, nor mutually exclusive with other recurrent genomic events in this tumor type. Indeed, the frequency of RB loss was significantly higher in HRR-deficient cases, and there was significant co-occurrence between RB and PTEN loss. By contrast, RB loss was a rare event in *CCNE1*g cases. Cases with RB or PTEN loss demonstrated reduced CN at their respective loci; however, not all cases with loss demonstrated low CN, suggesting mechanisms of inactivation beyond CN loss, consistent with reports of complex structural variants (SV) affecting both *RB1* and *PTEN* (6). Perhaps most interestingly, RB status discriminated outcome within the cases showing HRR gene aberrations, with the RB loss significantly associated with longer survival; RB loss did not significantly affect outcome in cases without identifiable events in HRR genes. It is unclear whether this phenotype is due to differences in therapy sensitivity, or whether concurrent RB loss results in HRR-aberrant tumors with more indolent behavior. Mechanistic work investigating the phenotypic and signaling consequences of RB loss in the context of HRR deficiency is now warranted, including investigation of the relative chemosensitivity of RB-lost and RB-intact HRR-deficient cells.

We present a large, pathologically confirmed HGSOC patient cohort with extensive follow-up and detailed clinical annotation, including chemotherapy response data. Together with the multiple layers of molecular characterization, these represent major strengths of this work. However, we were unable to characterize genome-wide SVs due to a lack of whole-genome sequencing, which is a limitation of the study. The lack of *BRCA1/2* SV and *BRCA1* promoter methylation data will have likely resulted in a more conservative HR estimate when comparing our HRR-aberrant and HRRwt populations. SVs such as translocations and inversions are known to affect *NF1* in a proportion of patients with HGSOC (6), and we were unable to characterize this patient group in our study. Future work should seek to provide an even greater resolution within the HRR-proficient patient population, including characterization of cases with *NF1* loss. We also acknowledge limitations in the nature of this cohort: our study benefits from an extensive follow-up period (median follow-up time 15 years); however, this necessitates the use of cases diagnosed prior to implementation of the most contemporary treatment modalities. Specifically, our cohort was not diagnosed in the era of maintenance antiangiogenic or PARPi use for first-line management, and a larger proportion of patients in our cohort underwent single-agent platinum rather than platinum-taxane doublet chemotherapy compared with contemporary cohorts. Moreover, most were treated within the era where achieving <1 cm RD was considered optimal debulking, and the rate of complete surgical resection is lower than that achieved in some modern tertiary centers.

Specifically regarding future stratification and rational trial design, our study highlights a number of major points. First, *CCNE1*g cases demonstrate poor outcomes despite underrepresentation in the most advanced-stage cases; this suggests inherently aggressive biology rather than a greater propensity for metastatic spread. Previous reports have suggested this may be due to intrinsic chemoresistance (44); however, we demonstrate no significant difference in first- and second-line response to chemotherapy. New treatment strategies are needed to improve survival of *CCNE1*g cases; clinical trials investigating agents targeting the cell cycle (such as WEE1 and ATR inhibitors) should focus on this patient group in the hope of improving outcomes in this underserved population.

Second, we highlight that overexpression of *EMSY* occurs in a group of *BRCA1*/*2* wild-type patients and is associated with a phenotype akin to HRR deficiency (improved survival and response to multiple lines of chemotherapy). This is consistent with EMSY's known role as a regulator of BRCA2 function (16); however, EMSY has received relatively little attention in HGSOC. This underinvestigated group warrants further attention as a patient population with potential HRR deficiency. Currently, it is unknown whether their HRR-deficient-like phenotype extends to exquisite PARP inhibitor sensitivity or whether these patients are identifiable by techniques such as genomic scarring assays.

Third, we highlight that RB and PTEN loss are frequent events in HGSOC. Tumors with disruption of RB may represent a subgroup more susceptible to cell cycle–targeted agents, such as WEE1 or ATR inhibitors; subgroup analysis, including identification of *CCNE1*g and RB loss, should be performed when assessing the efficacy of these agents. Similarly, those with loss of PTEN may represent the best candidates for agents targeting the PI3K/AKT pathway, many of which are currently under investigation in ovarian cancer (45). RB and PTEN events have previously been conceptualized to occur mutually exclusively to other events (46, 47); however, our data demonstrate that this is not the case. RB loss significantly co-occurs with HRR aberrations and we show that RB loss within this context is associated with even more favorable outcomes than HRR deficiency alone. Although a previous report has demonstrated enrichment for concurrent RB loss and HRR deficiency in long-term survivors, this was a study of heavily selected patients (48). We show that PTEN loss can co-occur with other events such as *BRCA1*/*2*m and that it significantly co-occurs with RB loss. This is important information for interpretation and design of trials of agents targeting PI3K/PTEN/AKT in ovarian cancer (45).

Lastly, and more generally, knowledge of potentially targetable molecular events that co-occur or demonstrate mutual exclusivity is crucial to inform future therapeutic strategies. We believe that our study demonstrates that the concept of a simple model where each HGSOC in a patient cohort is ascribed to a single key driver event is outdated. There are some subgroups (e.g., *CCNE1*g) where this may be appropriate, but for many tumors, multiple key events coexist and may independently affect outcome (as is seen for RB status in HRR-deficient patients).

Together, these data provide a high-resolution picture of the molecular landscape in HGSOC, integrating genomic sequencing with CN data, transcriptomic profiling, and immune cell infiltration burden in a cohort of HGSOC with rich clinical annotation. Specific transcriptomic subtypes are associated with marked differences in the frequency of HRR gene aberrations, *CCNE1*g and infiltration of immune cells; integration of these data highlights patient groups which may benefit most from conventional chemotherapy and specific targeted biological agents. Patients with *CCNE1*g and HRRwt tumors represent those with the greatest unmet clinical need; investigations of new treatment strategies should focus on this patient group. RB and PTEN loss is common in HGSOC and frequently occurs alongside other molecular events, with RB loss affecting a large number of tumors with HRR gene aberrations.

## Supplementary Material

Supplementary Data

Supplementary Data

Supplementary Data
